# Decrease in HBsAg After TAF Switching from Entecavir During Long-Term Treatment of Chronic Hepatitis B Virus Infection

**DOI:** 10.3390/v17010044

**Published:** 2024-12-31

**Authors:** Kazuto Tajiri, Yuka Hayashi, Aiko Murayama, Nozomu Muraishi, Masami Minemura, Ichiro Yasuda

**Affiliations:** Third Department of Internal Medicine, Faculty of Medicine, Academic Assembly, University of Toyama, 2630 Sugitani, Toyama 930-0194, Japan; yukaberry0822@yahoo.co.jp (Y.H.); aikokikuchi@me.com (A.M.); mnozomu1126@yahoo.co.jp (N.M.); minemura@med.u-toyama.ac.jp (M.M.); yasudaic@med.u-toyama.ac.jp (I.Y.)

**Keywords:** hepatitis B virus, tenofovir alafenamide fumarate, nucleos(t)ide analogue switching, HBsAg decrease, dyslipidemia

## Abstract

Achieving HBsAg seroclearance is a key goal in treating chronic hepatitis B virus (HBV) infection but remains difficult with nucleos(t)ide analogues (NAs). Tenofovir alafenamide fumarate (TAF), a recommended NA for managing chronic HBV infection (CHB), has uncertain effects on HBsAg levels and potential adverse events when used long-term after switching from entecavir (ETV). We retrospectively evaluated 77 CHB patients, including 47 who switched from ETV to TAF with a median follow-up of 40 months post-switch and a median of 60 months of HBsAg monitoring pre-switch. No significant change in HBsAg levels was observed in the overall cohort post-switch, consistent with the ETV continuation group. However, a significant decrease in HBsAg was noted in patients with HBsAg < 100 IU/mL at the time of switching. HBsAg loss occurred in three patients who switched to TAF. No adverse effects were observed, and TAF was well tolerated. The most significant factor associated with achieving HBsAg < 100 IU/mL was the Fib-4 index, a marker of liver fibrosis, at the time of switching. Switching from ETV to TAF is an effective strategy in CHB management, with hepatic inflammation potentially playing an essential role in achieving HBsAg decrease. Patients with increased Fib-4 index were significantly more likely to show decreased HBsAg. This finding suggests patients with mild to moderate fibrosis may respond better to TAF in terms of HBsAg reduction.

## 1. Introduction

Chronic hepatitis B (CHB) virus infection is a serious and life-threatening issue in Asia [1]. Globally, about 250 million people suffer from CHB [2]. It increases risks of liver cirrhosis and hepatocellular carcinoma (HCC) [3], making its management a major concern. Evaluation of HB virus surface antigen (HBsAg) is vital since HBsAg loss is related to remission without virological or clinical relapse following discontinuation of antiviral treatments [4]. The risk of HCC is significantly lower in patients who achieve HBsAg reduction compared to those who do not [5]. Sustained HBsAg loss post-completion of antiviral treatment is considered a functional cure of CHB and a therapeutic goal [6,7].

Nucleos(t)ide analogues (NA) effectively suppress viral replication by inhibiting reverse transcription. Recommended treatments for CHB in Japan include entecavir (ETV), tenofovir disoproxil fumarate (TDF), and tenofovir alafenamide fumarate (TAF) for their high efficacy against resistant mutations [8], although resistance may still emerge, particularly with ETV. While resistance is rare in lamivudine-naïve patients (~1%), it occurs in up to 50% of lamivudine-experienced patients with ≥5-years ETV treatment [9]. However, no resistance has been reported in patients with ≤10-years TDF [10] or ≤3-years TAF treatment [11].

NA treatments are also associated with adverse events. ETV can cause mitochondrial injury, leading to renal insufficiency, while TDF is linked to hypophosphatemia, glomerular dysfunction, and decreased bone mineral density [12]. TAF offers improved renal and bone safety [13,14]. However, it has been associated with potential cholesterol dysfunction, although this remains controversial [15,16].

Reducing HBsAg levels is a key goal in CHB management but achieving it using NA alone is challenging [17]. Tenofovir has shown potential to induce interferon λ more effectively than drugs like ETV, as nucleotide analogs tend to exhibit superior interferon λ activity compared to nucleoside analogs [18]. TAF, a prodrug of tenofovir, delivers higher concentrations of the active drug to hepatocytes at lower doses than TDF (25 mg/day vs. 300 mg/day, respectively). This has prompted investigations, especially in Japan, into the effectiveness of switching from ETV to TAF to reduce HBsAg levels while maintaining favorable safety profiles. Although some studies have reported that TDF or TAF reduces HBsAg levels more effectively than ETV [14,19,20,21], these findings remain controversial [22,23].

Given the need for long-term NA administration in CHB, both efficacy and adverse effects must be considered. Switching to TAF from another NA may be a promising strategy for CHB management, but long-term post-switch effects require investigation. Here, we conducted an observational cohort study to evaluate changes in HBsAg levels in CHB patients who switched to TAF from another NA, with an extended observation period.

## 2. Materials and Methods

### 2.1. Patients

**Figure 1:**
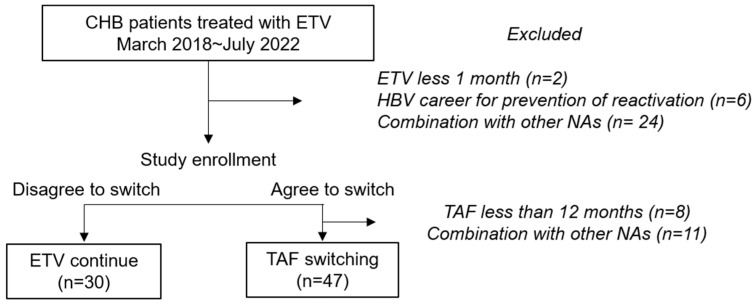
Study flowchart. CHB, chronic hepatitis B virus infection; ETV, entecavir; HBV, hepatitis B virus; NA, nucleos(t)ide analogues; TAF, tenofovir alafenamide fumarate.

We included CHB patients treated with ETV who switched to TAF between March 2018 and July 2022 at Toyama University Hospital. Definitions of inactive carrier, chronic hepatitis, cirrhosis, and CHB treatment followed Japanese guidelines [8]. Liver cirrhosis and hepatocellular carcinoma (HCC) were diagnosed by a hepatologist with >20-years’ experience. Liver fibrosis was assessed using Fib-4 index, an established marker for evaluating fibrosis progression in HBV patients [24], as previously described [25]. Serum HBsAg levels were measured using an Architect HBsAg QT assay (Abbott, North Chicago, IL, USA). Switches from another NA, such as ETV to TAF, followed established guidelines [8]. TAF was administered orally at a dose of 25 mg once daily after a meal. Patients with a <12-month observation period following TAF switching, <1 month of prior ETV administration, concurrent use of other NAs, or those who were HBV carriers receiving treatment solely for HBV reactivation prevention were excluded (Figure 1). Written informed consent from participants was waived due to the retrospective and non-interventional nature of the study, and an opt-out-choice was provided. The study adhered to the tenets of the Declaration of Helsinki. The study protocol was approved by Toyama University Hospital Institutional Ethics Committee (R2014096).

### 2.2. Patient Follow-Up and Adverse Event Monitoring

All patients were monitored every 1–3 months during ETV or TAF administration. Symptoms, physical examinations, tolerability, and laboratory results were recorded by clinicians. Treatment-related adverse events were graded using National Cancer Institute Common Terminology Criteria for Adverse Events (CTCAE) version 4.0. Renal function was assessed using an estimated glomerular filtration rate (eGFR, mL/min/1.73 m^2^) calculated from serum creatinine levels. Renal insufficiency was defined as eGFR < 60 in accordance with chronic kidney disease criteria [26]. Serum phosphate (P, mg/dL) levels were monitored, with a threshold of 2.5 mg/dL (Grade 2 per CTCAE), as the normal range is 2.8–4.5 mg/dL [27]. Serum total cholesterol (T-Cho, mg/dL) and low-density lipoprotein cholesterol (LDL-C, mg/dL) levels were measured post-switch. Cases with a ≥ 10% decline in eGFR, T-Cho, or LDL-C were evaluated. NA adherence was assessed from prescription records.

### 2.3. Change in HBsAg

HBsAg titers were monitored during ETV or TAF treatment. Since a low baseline HBsAg level (<100 IU/mL) predicts reduced risk of liver disease, including HCC and cirrhosis [28], we categorized and evaluated post-switch HBsAg levels into three groups: <100, 100–1000, and >1000 IU/mL. Pre-switch HBsAg levels were analyzed over a median of 60 (range: 3–60) months. Change in HBsAg levels was assessed using an annual change ratio, calculated by comparing change over 12 months, with a tolerance of ±3 months, during ETV or TAF treatment.

### 2.4. Statistical Analyses

Patient characteristics were summarized as medians and ranges and chronological data as mean ± standard deviation (SD). Categorical variables were compared using the chi-square or Fisher’s exact test as appropriate, and continuous variables using student’s *t*-test or the Mann–Whitney U test depending on data distribution. In multivariate analyses, variables with *p* < 0.10 were included. Results with *p* < 0.05 were deemed significant. We used SPSS version 19.0 (SAS Institute, Cary, NC, USA).

## 3. Results

### 3.1. Patient Characteristics

The present study included 47 CHB patients (male: 23, 48.9%) with a median age of 65 years, who were switched from ETV to TAF (Table 1). An additional 30 patients continued ETV administration. General organ function was largely preserved in both groups. Most patients were infected with HBV genotype C. Serum ALT and HBV-DNA levels were generally well-controlled (median ALT, 17 U/L and 15 U/L; undetectable HBV-DNA, 34/47 and 21/30 patients in the TAF-switch and ETV-continuation groups, respectively), as all participants had been treated with ETV for a median duration of 97 and 81 months, respectively (Table 1). Among patients with detectable HBV-DNA, two in the TAF-switch group had measurable HBV-DNA levels of 1.5 and 2.5 LogIU/mL, while one in the ETV-continuation group had a level of 1.8 LogIU/mL. In other cases, HBV-DNA signals were detected but not quantifiable in 11 and 8 patients in the TAF-switch and ETV-continuation groups, respectively. The median observation period following the switch to TAF was 40 months (range: 15–82 months).

### 3.2. HBV-DNA After TAF Switching

**Figure 2:**
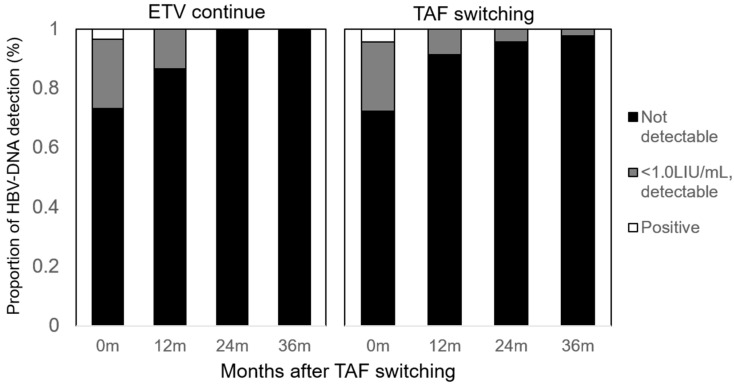
HBV-DNA levels after switching to TAF. The y-axis indicates the proportion (%) of HBV-DNA detection levels, and the x-axis represents time (months) since switching to TAF. Black bars show the proportion of patients with undetectable serum HBV-DNA, gray bars indicate those with detectable HBV-DNA below the quantification limit, and white bars represent patients with quantifiable HBV-DNA. The left panel shows presents data from the ETV continuation cohort, while the right panel shows data from the TAF switching cohort.

HBV-DNA was detected in 9 and 13 patients before switching to TAF. These cases showed a gradual decrease in HBV-DNA levels, becoming undetectable in all 24 patients by 24 months after the switch (Figure 2). Among cases with measurable HBV-DNA, levels decreased to half their initial values within 3 months (e.g., from 2.5 LogIU/mL before switching to 1.2 LogIU/mL at 3 months post-switch). The proportion of patients with detectable but unquantifiable HBV-DNA decreased from 29.4% before the switch to 12.5% at 36 months (Figure 2).

### 3.3. Adverse Effects and Tolerability After Switching to TAF

**Figure 3:**
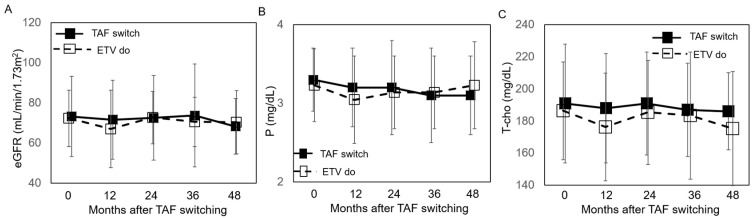
Changes after TAF switching. (**A**) Change in eGFR. The y-axis represents eGFR values. The x-axis represents time (months) since TAF switching. (**B**) Change in phosphorus (P)**.** The y-axis represents *p* values. The x-axis represents time (months) since TAF switching. (**C**) Change in total cholesterol (T-Cho). The y-axis represents T-Cho values. The x-axis represents time (months) since TAF switching. Each plot shows the mean values for study participants over time. Black squares and solid lines represent data from the TAF-switch group, while white squares and dotted lines represent data from the ETV-continuation group.

No serious adverse symptoms were reported after switching to TAF (Table 2). All patients adhered to the TAF regimen throughout the observation period, whereas a few patients (3/30, 10.0%) in the ETV continuation group demonstrated incomplete adherence. Regarding laboratory findings, no significant changes in eGFR were observed over the 5 years following the switch to TAF or continued ETV treatment (Figure 3A). When patients were stratified by an eGFR threshold of 60 mL/min/1.73 m^2^, which indicates decreased renal function, eGFR remained stable regardless of renal function status. However, more patients with decreased eGFR were included in the TAF-switch group compared to the ETV-continuation group (13 vs. 3 patients, respectively) (Supplementary Figure S1). Serum phosphorus levels (P) showed no significant changes after switching to TAF (Figure 3B), regardless of baseline serum P levels (Supplementary Figure S2). Similarly, serum cholesterol and LDL-cholesterol levels remained stable after switching to TAF (Figure 3C and Supplementary Figure S3).

### 3.4. Changes in HBsAg After TAF Switching

**Figure 4:**
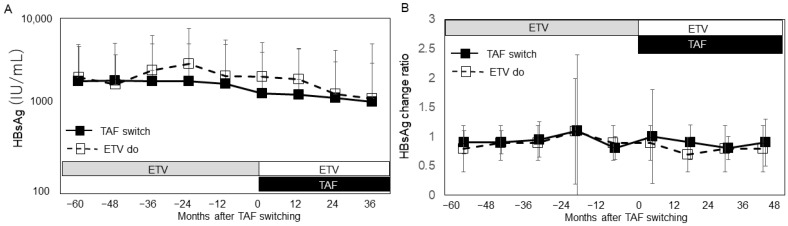
Changes in HBsAg before and after TAF switching. (**A**) Chronological change in HBsAg levels. The y-axis indicates HBsAg titers (IU/mL), and the x-axis indicates time (months) before and after TAF switching. The gray columns indicate the treatment period with entecavir (ETV), while the white and black columns indicate the period after switching to TAF. (**B**) Annual HBsAg change ratio during the observation period. The HBsAg change ratio was calculated by comparing annual changes in HBsAg levels. The gray columns represent the ETV treatment period, while the white and black columns represent the period after switching to TAF. In both panels, black squares and solid lines represent data from the TAF-switch group, while white squares and dotted lines represent data from the ETV continuation group.

**Figure 5:**
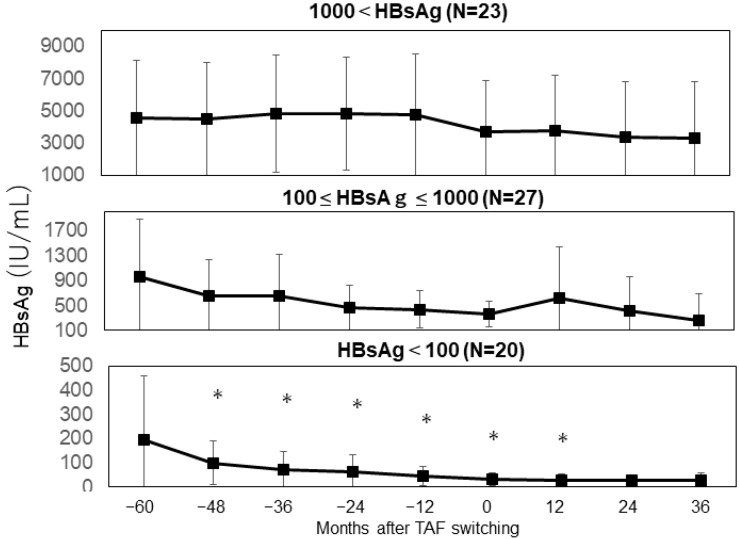
Changes in serum hepatis B-virus surface antigen (HBsAg) levels after TAF switching. The y-axis represents HBsAg levels (IU/mL), and the x-axis represents time (months) before and after switching to TAF. The top panel shows mean HBsAg levels in patients with HBsAg > 1000 IU/mL. The middle panel show mean levels in patients with HBsAg ranging from 100 to 1000 IU/mL, while the panel shows mean levels in patients with <100 IU/mL. Asterisks (*) denote statistically significant differences (*p* < 0.05).

Overall, serum HBsAg levels remained relatively stable, with a gradual decline observed over the 5 years following the switch to TAF or continued ETV treatment (Figure 4A,B). However, in patients with baseline HBsAg levels ≤ 100 IU/mL, a significant decrease in HBsAg levels was noted (Figure 5). Consequently, HBsAg loss was observed in three patients. To evaluate the effect of TAF switching, we also assessed changes in HBsAg levels prior to the switch. Over 5 years, HBsAg levels demonstrated a decreasing trend, although the changes were not statistically significant (Figure 4A,B). A decrease in HBsAg levels was observed exclusively in patients with baseline HBsAg levels < 100 IU/mL at the time of the switch. In contrast, no significant changes were observed in patients with HBsAg levels ≥ 100 IU/mL (Figure 5). Further analysis of factors associated with achieving HBsAg levels < 100 IU/mL revealed that the Fib-4 index was an independent contributing factor (Table 3).

## 4. Discussion

HBsAg loss remains the ultimate goal in CHB treatment, but achieving it is challenging with NA treatment alone. Additionally, long-term NA treatment necessitates consideration of accompanying conditions or adverse events, such as renal dysfunction and bone mineral loss. TAF, a prodrug of tenofovir, has shown potential for lowering HBsAg with fewer adverse effects on kidneys and bones. In this study, we investigated long-term changes in HBsAg levels and adverse events following the switch from ETV to TAF.

Our findings confirmed the HBV-DNA-lowering effects of TAF. Its antiviral efficacy is comparable to that of TDF, as demonstrated in a randomized trial with 96 weeks of observation [13]. TDF has shown superior virological responses in both NA-naïve [29] and NA-experienced or resistant HBV patients [30,31,32]. It has also proven effective and safe for pregnant women [33], with similar effects expected for TAF. Furthermore, TDF has demonstrated potential for reducing HCC risk. Large cohort studies have reported a reduced risk of HCC with TDF compared to ETV [5,18], although this remains controversial [34]. A recent randomized trial involving 148 CHB-HCC patients showed that TDF reduced recurrence risk after curative surgical resection compared to ETV [35]. Overall, the antiviral effectiveness of TAF treatment is promising, and its potential to reduce HCC risk should be explored in future studies.

TAF also offers favorable renal and bone safety compared to TDF [36,37,38]. For example, TDF treatment in HIV patients has been associated with hypophosphatemia and glomerular dysfunction in 20% of cases [39]. In CHB patients, TDF led to a −3 mL/min decline in eGFR and a −2% decrease in bone mineral density at 96 weeks compared to TAF [13]. Conversely, switching to TAF from ETV has been linked to improved eGFR in retrospective cohorts [15,40], although no such improvement was observed in a prospective cohort [41]. In our study, eGFR was maintained in both cohorts. Despite including more patients with decreased baseline GFR in the TAF switched cohort, renal function remained stable, suggesting renal protective effects of TAF. Hypophosphatemia remained unchanged in a retrospective cohort [15], but bone mineral density improved in a prospective cohort [41]. In this study, hypophosphatemia slightly improved in the TAF-switched cohort, suggesting a favorable effect of TAF on bone health. A systematic review on dyslipidemia during TAF treatment indicated worsening dyslipidemia compared to TDF in patients with a history of diabetes or hypertension [16]. Therefore, careful consideration may be required when switching from TDF to TAF in patients with metabolic conditions. In our study, no decrease in eGFR, progression of hypophosphatemia, or worsening of dyslipidemia was observed. Furthermore, good tolerability was confirmed in our cohort, consistent with previous findings [20]. These results indicate that TAF demonstrates good tolerability and safety profiles over more than 3 years after switching from ETV.

In this study, HBsAg loss was observed in some participants. Long-term observation revealed a decrease in HBsAg levels, which did not appear to be directly related to the switch to TAF, as no significant changes in the rate of decrease were observed before and after the switch. Previous studies with extended observation periods have reported a slight reduction in HBsAg levels during TAF treatment, ranging from −0.1 to −0.2 log IU/mL per year [15,19]. Our findings indicated that the decrease in HBsAg levels was greater in patients with HBsAg < 100 IU/mL, with Fib-4 index emerging as a contributing factor. The complex relationships between liver fibrosis progression, HBV viral load, and hepatocarcinogenesis have been reported [24,42,43]. A high Fib-4 index has been identified as a predictive marker of HCC in HBV-infected patients, as sustained liver inflammation and subsequent liver fibrosis are well-established risks for HCC [42]. Conversely, a non-linear association between HBV viral load and HCC risk has also been noted [24,43]. It is hypothesized that optimal hepatic inflammation promotes HBV-DNA reduction, whereas prolonged inflammation leads to liver fibrosis progression. This may explain our finding that Fib-4 index contributed to the observed HBsAg reduction. Importantly, no adverse effects were observed in association with the decrease in HBsAg levels after switching to TAF. Tenofovir has been shown to induce interferon-lambda3 in intestinal mucosal cells and increase interleukin-12p70 and tumor necrosis factor-alpha in peripheral mononuclear cells, mechanisms that may contribute to HBsAg reduction [44,45]. While the long-term effects of nucleos(t)ide analogs are time-dependent, some additive HBsAg-lowering effects with TAF may exist. Further studies are required to elucidate the mechanisms leading to HBsAg levels < 100 IU/mL.

This study had several limitations. First, the small sample size limited the statistical significance of the findings. Additionally, the single-arm trial design did not allow for a definitive assessment of TAF’s effects without comparison to crossover or control arms. Furthermore, similar works about TAF switching have been already found. Nevertheless, the long-term observation of HBsAg changes before and after switching to TAF, including adverse events, offers valuable insights for clinicians. Second, detailed evaluations of adverse events were not performed, including assessments of glomerular or tubular function and bone mineral density. Comprehensive lipid profile analyses were also not conducted. Future studies should incorporate these evaluations to provide a more thorough understanding of potential adverse events. Third, more sensitive markers, such as HBV-RNA and HB core-related antigen, were not analyzed, nor were advanced HBsAg assays like iTACT HBsAg. Investigating these markers in future studies could yield deeper insights.

## 5. Conclusions

This study demonstrated changes in HBsAg levels and adverse events over a long-term observation period following the switch from ETV to TAF. A significant decrease in HBsAg was observed exclusively in patients with HBsAg < 100 IU/mL, with no negative effects attributed to TAF. Additionally, no adverse events, including dyslipidemia, were noted. These findings suggest that switching to TAF is a viable and promising strategy for managing CHB.

## Figures and Tables

**Table 1:** Patient characteristics.

	Median (Range); N/N, Numbers of Cases		
	ETV Continue	TAF Switching	*p*
Age (years)	65 (44–75)	64 (43–80)	0.88
Gender (male/female)	21/9	23/24	0.10
Genotype (A/B/C/ND)	1/4/10/15	1/7/12/20	
ALT (U/L)	15 (8–55)	17 (8–32)	0.11
Platelets (×10^3^/mm^3^)	19.5 (7.8–41.7)	17.7 (4.7–34.6)	0.44
eGFR (mL/min/1.73 m^2^)	70.1 (51.0–100.6)	73.2 (40.3–138.6)	0.62
Phosphorus (mg/dL)	3.2 (2.2–4.1)	3.2 (2.3–4.2)	0.66
Total cholesterol (mg/dL)	182 (142–250)	193 (121–310)	0.59
LDL-cholesterol (mg/dL)	111 (55–147)	112 (72–135)	0.98
HBeAg (positive/negative/ND)	2/9/19	2/11/34	
HBsAg (IU/mL)	538 (1.14–9077)	393 (0.46- > 10,000)	0.52
HBV-DNA (positive/not detected)	9/21	13/34	0.84
Fib-4 index	1.69 (0.77–11.91)	1.87 (0.56–7.73)	0.34
Cirrhosis (+/−)	5/25	12/35	0.36
HCC (+/−)	6/24	7/40	0.56
Duration from HBeSC (months)	102 (−90–250)	92 (−10–1460)	0.63
Duration of prior ETV (months)	81(1.0–150)	97 (29–268)	0.02

ETV, entecavir; TAF, tenofovir alafenamide fumarate; ND, not determined; ALT, alanine amino transferase; eGFR, estimated glomerular filtration rate; LDL, low-density lipoprotein; HBeAg, hepatitis B envelope antigen; HBsAg, hepatitis B surface antigen; HCC, hepatocellular carcinoma; HBeSC, hepatitis B envelope seroconversion.

**Table 2:** Treatment-related adverse events and tolerability.

	Numbers of Cases (%)		
	ETV Continue	TAF Switching	*p*
Serious AEs (≥Grade 3)	0 (0)	0 (0)	1.00
AEs leading to discontinuation	0 (0)	0 (0)	1.00
eGFR decrease (>10%)	12 (40.0)	13 (27.7%)	0.32
Hypophosphatemia (≥Grade 2)	3 (10.0)	5 (10.6)	1.00
Cholesterol increase (>10%)	9 (30.0)	9 (19.1)	0.29
LDL-cholesterol increase (>10%)	2 (6.6)	7 (14.8)	0.47
Bone fracture	0 (0)	0 (0)	1.00
ALT increase (≥Grade 1)	2 (6.6)	3 (6.4)	1.00
HBV-DNA increase (>1 LogIU/mL)	0 (0)	0(0)	1.00
Emergence of resistant mutant	0 (0)	0 (0)	1.00
NAs adherence <80% dose intensity	0 (0)	0 (0)	1.00
<95% dose intensity	3 (10.0)	0 (0)	0.06

ETV, entecavir; TAF, tenofovir alafenamide fumarate; AEs, adverse events; eGFR, estimated glomerular filtration rate; LDL, low-density lipoprotein; ALT, alanine aminotransferase.

**Table 3:** Contributing factors to HBsAg < 100 IU/mL.

	Univariate			Multivariate		
	OR	95% CI	*p*	OR	95% CI	*p*
Age	1.064	1.009–1.121	0.021	1.047	0.990–1.108	0.108
Platelet	0.941	0.873–1.015	0.114			
Fib-4 index	1.741	1.113–2.722	0.015	1.583	1.032–2.426	0.035
Duration from initial NA	0.998	0.991–1.006	0.666			
Duration from HBeSC	1.000	0.997–1.003	0.827			
TAF switching	1.422	0.548–3.691	0.469			

HBsAg, hepatitis B virus surface antigen; OR, odds ratio; CI, confidence interval; NA, nuculeos(t)ide analogue; HBeSC, hepatitis B virus envelope antigen seroconversion; TAF, tenofovir alafenamide fumarate.

## Data Availability

The data from this study are available from the corresponding author upon reasonable request.

## Supplementary Materials

The following supporting information can be downloaded at https://www.mdpi.com/article/10.3390/v17010044/s1: Figure S1. Changes in eGFR. Change in patients with eGFR ≥ 60 are shown in the upper panel, whereas changes in those with eGFR < 60 are shown in the lower panel. Figure S2, Changes in serum phosphorus. Changes in patients with *p* > 2.5 are shown in the upper panel, whereas changes in those with *p* ≤ 2.5 are shown in the lower panel. Figure S3, Changes in LDL-cholesterol. The black square and solid lines represent the data for the TAF-switch group, whereas the white square and dotted lines represent the data for the ETV-continuation group.
