# Supplementary figures and images for: Decrease in HBsAg After TAF Switching from Entecavir During Long-Term Treatment of Chronic Hepatitis B Virus Infection
Source: Viruses. 2024 Dec 31;17(1):44. doi: 10.3390/v17010044 (PMC11769490; doi:10.3390/v17010044)

Supplementary Figure S1.

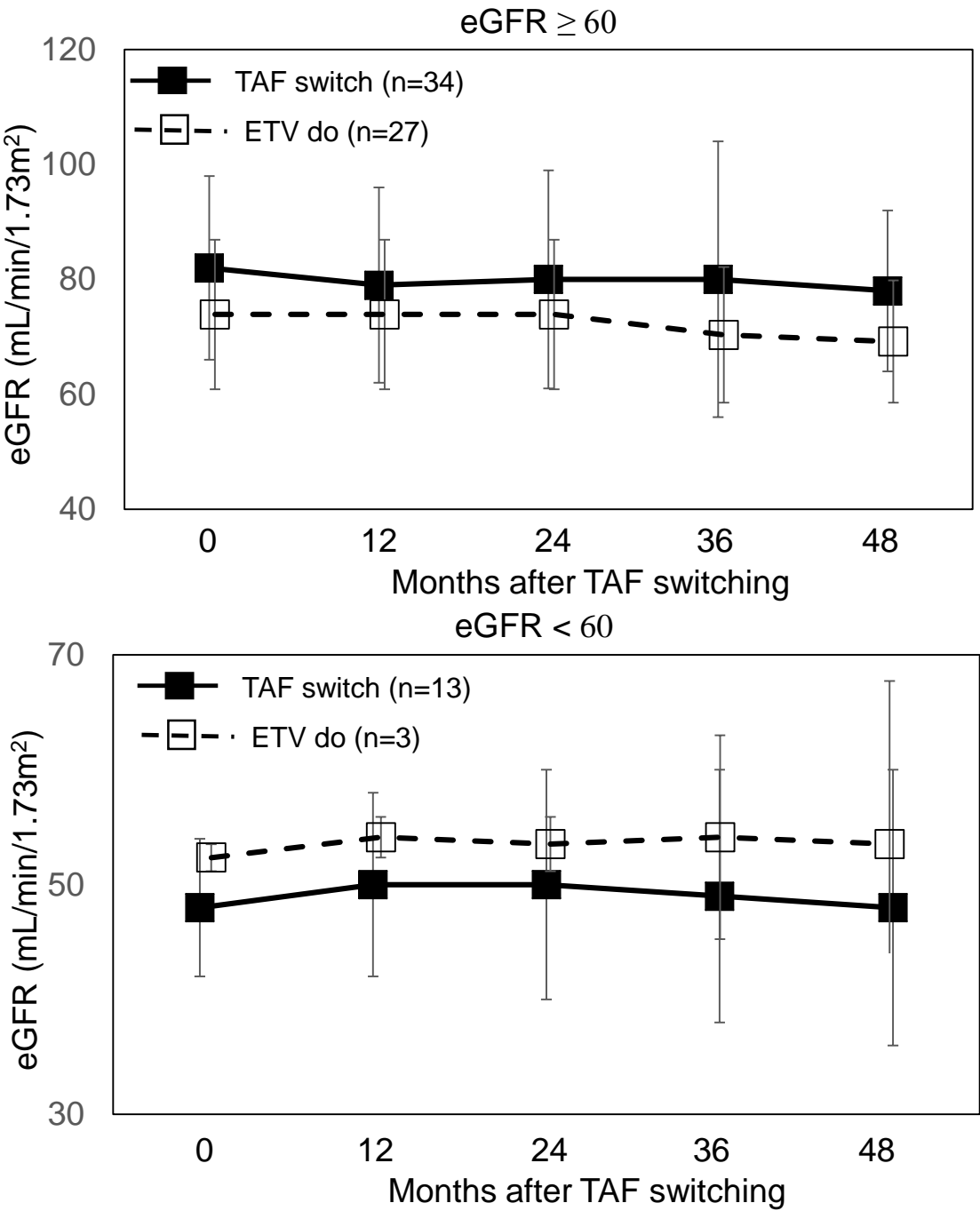

Supplementary Figure S2.

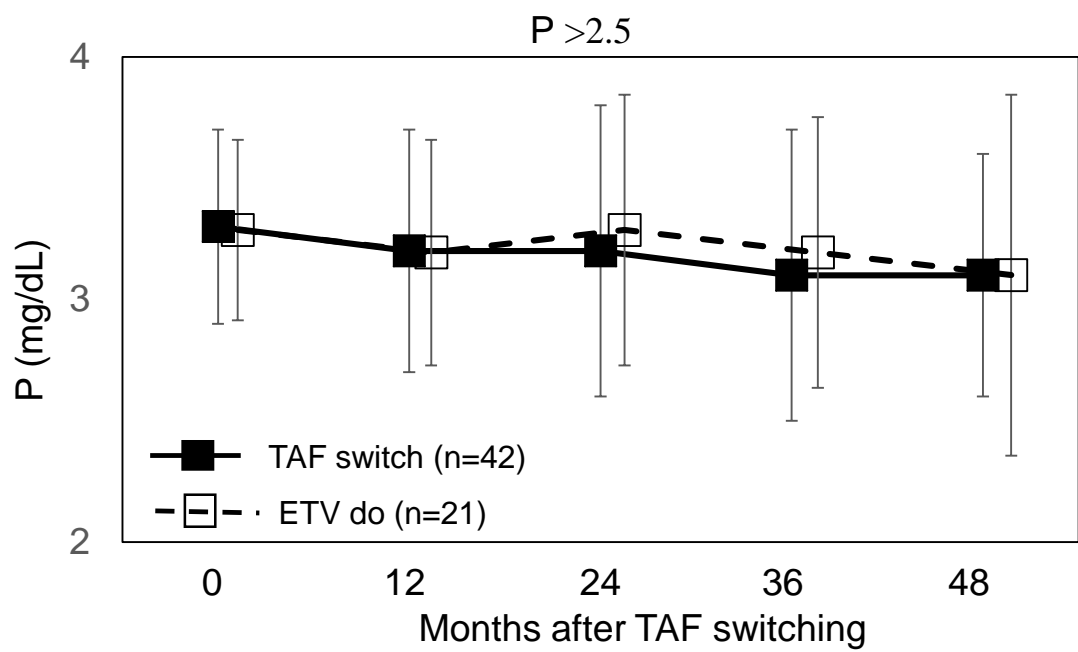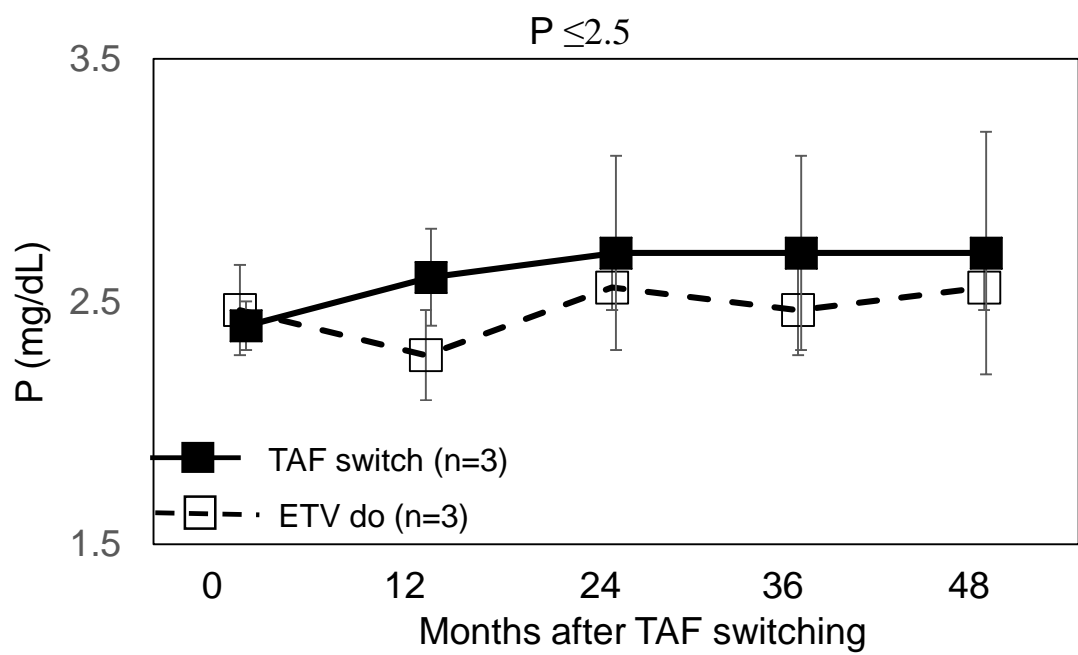

Supplementary Figure S3.

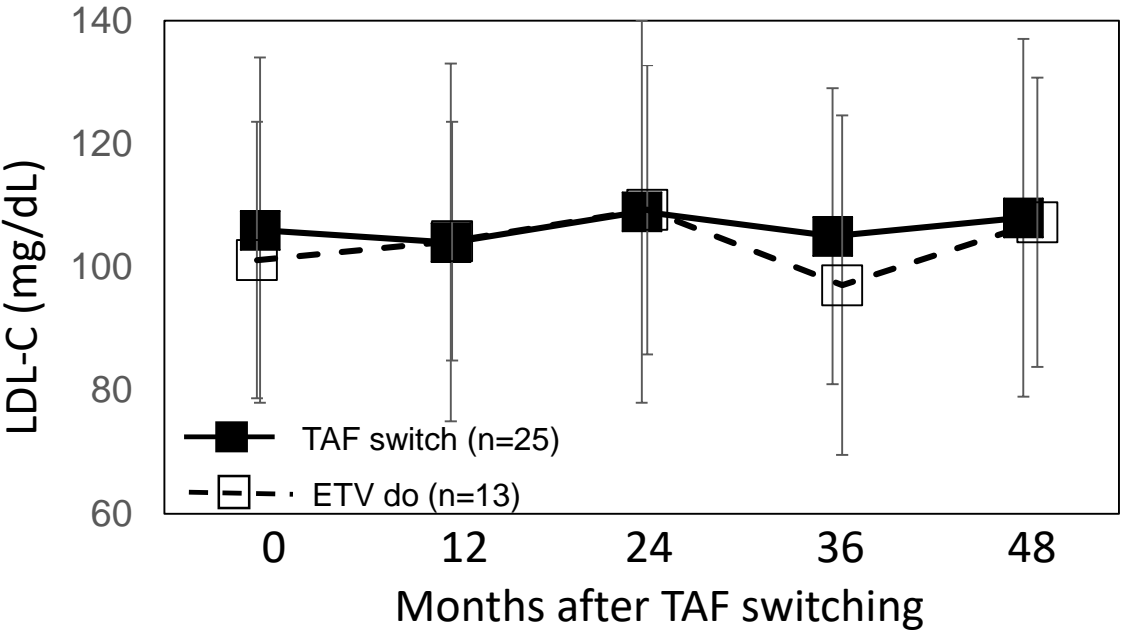

Supplement: Supplementary file 1 [file viruses-17-00044-s001.zip › viruses-3371006-supplementary.pdf]
